# *Armillaria mellea* Mycelia Alleviate PM2.5-Induced Pulmonary Inflammation in Murine Models

**DOI:** 10.3390/antiox13111381

**Published:** 2024-11-12

**Authors:** Yi-Ping Huang, Yu-Tsen Huang, Hui-Yu Wu, Li-Fang Chou, You-Shan Tsai, Yih-Min Jiang, Wan-Ping Chen, Ting-Wei Lin, Chin-Chu Chen, Chih-Ho Lai

**Affiliations:** 1 Department of Physiology, School of Medicine, China Medical University, Taichung 404333, Taiwan; 2 Department of Microbiology and Immunology, School of Medicine, China Medical University, Taichung 404333, Taiwan; 3 Department of Microbiology and Immunology, Graduate Institute of Biomedical Sciences, Chang Gung University, Taoyuan 333323, Taiwan; 4 Kidney Research Center, Chang Gung Memorial Hospital at Linkou, Taoyuan 333423, Taiwan; 5 Biotech Research Institute, Grape King Bio, Taoyuan 325002, Taiwan; 6 Institute of Food Science and Technology, National Taiwan University, Taipei 106216, Taiwan; 7 Department of Food Sciences, Nutrition, and Nutraceutical Biotechnology, Shih Chien University, Taipei 104336, Taiwan; 8 Department of Bioscience Technology, Chung Yuan Christian University, Taoyuan 320314, Taiwan; 9 Department of Nursing, Asia University, Taichung 413305, Taiwan; 10 Research Center for Emerging Viral Infections, Institute of Immunology and Translational Medicine, Chang Gung University, Taoyuan 333323, Taiwan; 11 Molecular Infectious Disease Research Center, Chang Gung Memorial Hospital at Linkou, Taoyuan 333423, Taiwan

**Keywords:** *Armillaria mellea* mycelia, PM2.5, macrophage, pulmonary inflammation

## Abstract

Particulate matter (PM) with an aerodynamic diameter of ≤2.5 μm (PM2.5) significantly contributes to various disease-related respiratory inflammations. *Armillaria mellea*, recognized for its medicinal properties, could alleviate these respiratory ailments. However, its efficacy against PM2.5-induced inflammation remains elusive. In this study, we investigated whether *A. mellea* mycelia could mitigate PM2.5-induced respiratory inflammation and assessed the underlying mechanisms. Our results showed that *A. mellea* mycelia significantly reduced PM2.5-induced nitric oxide (NO) production and nuclear factor (NF)-κB activation in macrophages. Furthermore, *A. mellea* mycelia suppressed the expression of inflammatory mediators, indicating their potent antioxidant and anti-inflammatory properties. In murine models, *A. mellea* mycelia mitigated PM2.5-induced lung inflammation and cytokine secretion, restoring lung inflammatory status. Our results highlight the potential of *A. mellea* mycelia to treat PM2.5-induced respiratory inflammation. The antioxidant and anti-inflammatory effects of *A. mellea* mycelia demonstrated in vitro and in vivo hold promising potential for developing respiratory health improvement interventions upon PM2.5 exposure.

## 1. Introduction

Particulate matter (PM) with an aerodynamic diameter of ≤2.5 μm (PM2.5) is widely recognized for its role in inducing respiratory inflammation [1,2]. Primary PM2.5 sources include vehicle and industrial emissions, as well as wildfires [3]. Prolonged PM2.5 exposure is reportedly linked to an increased risk of respiratory diseases such as asthma, chronic obstructive pulmonary disease, and lung cancers [4,5,6]. Several countries maintain monitoring networks to assess environmental PM2.5 concentrations. Nevertheless, an annual escalation in PM2.5 pollution was registered, especially in regions with significant industrial development [7].

Exposure to PM2.5 could lead to immune cell infiltration and the activation of inflammatory pathways, particularly influencing the alveolar macrophage functions [8,9]. Such inflammatory pathway activation is linked to the oxidative stress response elicited by PM2.5 exposure in the respiratory tract [10]. Notably, oxidative stress assumes a pivotal role in the detrimental impacts and mechanisms of PM2.5 on the respiratory system, eliciting diverse injurious pathways that culminate in inflammation and cellular damage [11]. Since low-level exposure to PM2.5 poses certain public health risks [12], mitigating PM2.5-induced pulmonary inflammation is crucial for human health. Various natural products and their constituents have been investigated for their potential to alleviate PM2.5-induced pulmonary injury and enhance lung function. Suppressing oxidative stress provides benefits in alleviating PM2.5-induced systemic dysfunction [13,14,15]. For instance, *Panax ginseng* (black ginseng), a traditional herb, reportedly exerts beneficial effects against PM2.5-induced lung endothelial cell barrier disruption and reactive oxygen species (ROS) generation, thus attenuating pulmonary inflammation [16]. *Securinega suffruticosa*, a plant species native to Asia and Europe, mitigates PM2.5-induced lung and vascular inflammation by inhibiting ROS and downregulating the NOD-like receptor pyrin domain containing-3 (NLRP3) inflammasome signaling pathway [17]. Despite the identification of numerous herbal medicines with the potential to alleviate PM2.5-induced lung injury, their underlying mechanisms vary and require further investigation.

*Armillaria mellea* is a mushroom with recognized medicinal properties, including immunomodulatory, anti-inflammatory, and pulmonary protective attributes [18]. *A. mellea* reportedly exhibited antioxidant activity [19,20], potentially alleviating respiratory symptoms resulting from exposure to air pollution. Mycelia from medicinal mushrooms contain a variety of constituents that offer numerous biological benefits to human health [21]. However, the capacity of *A. mellea* mycelia to ameliorate PM2.5-induced respiratory inflammation remains unclear. In this study, we aimed to elucidate whether submerged cultured *A. mellea* mycelia can mitigate the adverse effects of PM2.5 on the airways and assess the underlying mechanisms for improving respiratory inflammation. Using rigorous experimental methodologies, including in vivo and in vitro assessments, we aimed to elucidate the therapeutic potential of *A. mellea* mycelia against PM2.5-induced respiratory ailments.

## 2. Materials and Methods

### 2.1. Cell Culture

RAW264.7 cells (ATCC TIB-71), a macrophage cell line, were maintained in Dulbecco’s Modified Eagle Medium (DMEM, Invitrogen, Carlsbad, CA, USA) supplemented with 10% complement-inactivated fetal bovine serum (HyClone, Logan, UT, USA) at 37 °C in a 5% CO_2_ atmosphere [9].

### 2.2. Preparation of Ethanol Extracts of A. mellea Mycelia

The ethanol extracts of *A. mellea* mycelia were prepared according to previously established procedures [22]. For 10 d, *A. mellea* was cultured on potato dextrose agar slants at 25 °C. The *A. mellea* mycelia were then transferred into 1 L of synthetic culture medium (consisting of 2% glucose, 1% soybean powder, 0.1% yeast extract, and 0.1% peptone, pH 4.0) and were incubated at 25 °C for an additional 10 d with mild shaking. Subsequently, the fermentation process was scaled up in 20-ton fermenters for 10 d. The fermented broth was harvested, lyophilized, ground into a powder, and subjected to ethanolic extraction (using a ratio of 1:40 *w*/*v* in 95% ethanol). The ethanolic suspension was sonicated overnight and was then centrifuged at 15,000× *g* for 1 h. The resulting supernatant was subjected to vacuum concentration, which effectively removed ethanol and water from the final extract. The prepared extract was then condensed into a paste for use in subsequent experiments. We then performed high-performance liquid chromatography (HPLC) to identify and characterize the key components of *A. mellea* mycelia extract. The analysis revealed that the major constituents of *A. mellea* mycelia extract are Armillaridin (retention time: 19.6 min) and Melledonal C (retention time: 10.1 min), detected at a wavelength of 254 nm (Figure S1).

### 2.3. Preparation of PM2.5

Particulate matter with a diameter smaller than 2.5 μm (PM2.5) (RM8785) was purchased from the National Institute of Standards and Technology (Gaithersburg, MD, USA) [23]. PM2.5 was added to Dulbecco’s Modified Eagle Medium (DMEM) at specified concentrations for subsequent experiments, as described in our previous study [9].

### 2.4. Macrophage Phagocytosis Assay

The Phagocytosis Assay Kit (IgG FITC) from Cayman Chemical (Ann Arbor, MI, USA) was employed to assess the impact of PM2.5 on macrophage phagocytic activity, as described previously [9]. In brief, RAW264.7 cells were exposed to PM2.5 (30 μg/mL) for 24 h, followed by incubation with latex beads coated with fluorescent-labeled rabbit IgG for an additional 4 h. Subsequently, the cells were fixed using 4% paraformaldehyde, and flow cytometry (Becton Dickinson, San Jose, CA, USA) was utilized to analyze the phagocytic activity.

### 2.5. Western Blot Assay

The protein expression levels of iNOS, COX-2, and β-actin were assessed using a Western blot assay. RAW264.7 cells were treated with ethanol extracts of *A. mellea* mycelia, followed by exposure to PM2.5 (30 μg/mL) for 24 h. Cell lysates were prepared in 100 μL of RIPA buffer (Roch, Indianapolis, IN, USA) and subjected to Western blot analysis. The samples were separated by 10% SDS-PAGE and then transferred onto polyvinylidene difluoride membranes (Millipore, Billerica, MA, USA). Following blocking with 5% skim milk, the membranes were incubated with primary antibodies specific against inducible nitric oxide synthase (iNOS) and cyclooxygenase-2 (COX-2) (Abcam, Boston, MA, USA), respectively. The membrane was then probed with horseradish peroxidase (HRP)-conjugated secondary antibodies (Millipore). The protein of interest was visualized using ECL Western Blotting Detection Reagent (BIOMAN, Taipei, Taiwan) and analyzed with an Azure C400 system (Azure Biosystems, Dublin, CA, USA) [24].

### 2.6. Analysis of Nitric Oxide (NO) and Cytokine Production

RAW264.7 cells were treated with ethanol-extracted *A. mellea* mycelia and exposed to PM2.5 (30 μg/mL) for 24 h. The culture supernatant was collected for the determination of the NO concentration using Griess reagent (Sigma-Aldrich, St. Louis, MO, USA) [25]. The concentrations of a high mobility group box 1 (HMGB1), interleukin 1β (IL-1β), interferon γ-induced protein 10 (IP-10), and keratinocyte-derived cytokine (KC) were determined using a sandwich enzyme-linked immunosorbent assay (R&D Systems, Minneapolis, MN, USA) [26].

### 2.7. NF-κB Luciferase Activity Assay

RAW264.7 cells were transfected with an NF-κB-luciferase reporter construct and subsequently treated with ethanol-extracted *A. mellea* mycelia before exposure to PM2.5 (30 μg/mL). Following a 24 h incubation period, cell lysates were prepared for a luciferase assay using the Dual-Luciferase Reporter Assay System (Promega, Madison, WI, USA). Co-transfection with a β-galactosidase expression vector (Promega) was conducted to normalize the reporter gene assay [27]. 

### 2.8. Implementation of Animal Study

Male BALB/c mice (6 weeks old) were purchased from the National Laboratory Animal Center (Taipei, Taiwan) in adherence with the Animal Care and Use Guidelines for Chang Gung University. The experimental protocol was approved by the Institutional Animal Care and Use Committee (IACUC Approval No.: CGU108-145). The mice were divided into four groups (with six mice per group): (i) PBS (mock), (ii) PM2.5-exposed, (iii) *A. mellea* mycelial extract-treated, and (iv) a combination of PM2.5-exposed with *A. mellea* mycelial extract-treated. PM2.5 (25 μg per mouse) was administered by intratracheal instillation twice weekly for a total of eight times over four weeks (totaling 200 μg). PBS or *A. mellea* mycelial extract (1.5 mg per mouse) was administered through oral gavage once daily for a total duration of 27 days. On day 28, blood samples were collected from mouse tail veins using 25-gauge needles for all tested groups. Serum was isolated by centrifugation at 1000× *g* for 3 min at room temperature. The mice were euthanized, and bronchoalveolar lavage fluid (BALF) and lungs were isolated following established protocols, as previously described [9]. Briefly, the trachea was flushed with normal saline, and the collected fluid was centrifuged at 500× *g* for 5 min at 4 °C. Cytokine levels in the supernatants were then analyzed using ELISA.

### 2.9. Immunohistochemistry (IHC) Analysis

Murine lung tissues were processed for hematoxylin–eosin (H&E) or immunohistochemistry (IHC) staining following established protocols, as detailed in prior work [9]. The lung sections were immunostained with antibodies targeting interleukin 1-beta (IL-1β) (ab9722, Abcam) and tumor necrosis factor-α (TNF-α) (ab6671, Abcam), respectively, followed by incubation with horseradish peroxidase (HRP)-conjugated secondary antibody (D39-110, OriGene, Rockville, MD, USA), and developed using the Avidin-Biotin Complex Kit (ABC Kit, Vector Laboratories, Burlingame, CA, USA). Subsequently, the stained tissues were examined and analyzed using a microscope (AXIO IMAGER M2, Carl Zeiss, Oberkochen, Germany). Inflammatory cell infiltration in the lungs was assessed using the following criteria: score 0 for normal lung tissue, score 1 for mild inflammation, score 2 for moderate inflammation, and score 3 for severe inflammation.

### 2.10. Statistical Analysis

Statistical significance between the two groups was determined using Student’s *t*-test or the Mann–Whitney U test. Comparisons involving more than two groups were evaluated using one-way ANOVA with Tukey’s post hoc test. A *p*-value less than 0.05 was considered statistically significant. The figures were generated using Prism Program (v.9.0.0, GraphPad, San Diego, CA, USA).

## 3. Results

### 3.1. A. mellea Mycelia Mitigate PM2.5-Induced Nitric Oxide (NO) Production in Macrophages

**Figure 1:**
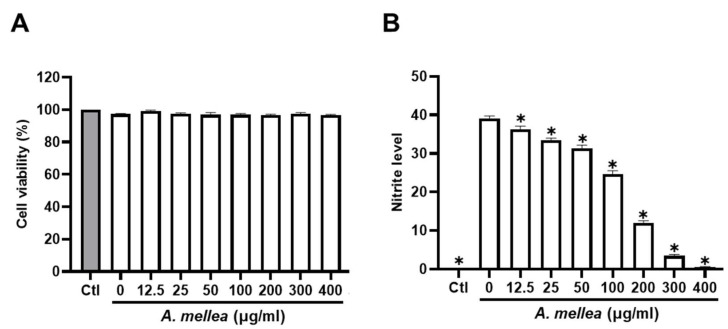
*A. mellea* mycelia diminish PM2.5-induced nitric oxide production in macrophages. RAW264.7 cells were pretreated with varying concentrations of *A. mellea* mycelial extract (0–400 μg/mL) before exposure to PM2.5 (30 μg/mL) for 24 h. (**A**) Cell viability was evaluated using the MTT assay. (**B**) Nitric oxide production in the culture supernatant was quantified by measuring nitrite levels with Griess reagent. The data were presented as the mean ± standard deviation from three independent experiments. Statistical significance was determined using one-way ANOVA with Tukey’s post hoc test. *, *p* < 0.05 compared to *A. mellea*-untreated group.

We initiated our investigation by assessing the impact of PM2.5 exposure on macrophage viability and NO production, exposing RAW264.7 macrophages to varying (0–30 μg/mL) PM2.5 concentrations for 24 h. Our results revealed that exposure to 30 μg/mL PM2.5 did not elicit significant changes in cell viability yet notably increased NO production (Figure S2). Consequently, we selected the PM2.5 concentration of 30 μg/mL for our subsequent experiments. To further elucidate the potential anti-inflammatory properties of the ethanol extracts of *A. mellea* mycelia, we assessed cytotoxicity and NO production using the macrophage models. As shown in Figure 1A, the *A. mellea* mycelial extract at 400 μg/mL did not significantly impact cell viability. The concentrations between 20 and 400 μg/mL significantly and concentration-dependently suppressed the PM2.5-induced NO production in macrophages (Figure 1B).

### 3.2. A. mellea Mycelia Alleviate PM2.5-Associated Inflammatory Mediator Effects in Macrophages

**Figure 2:**
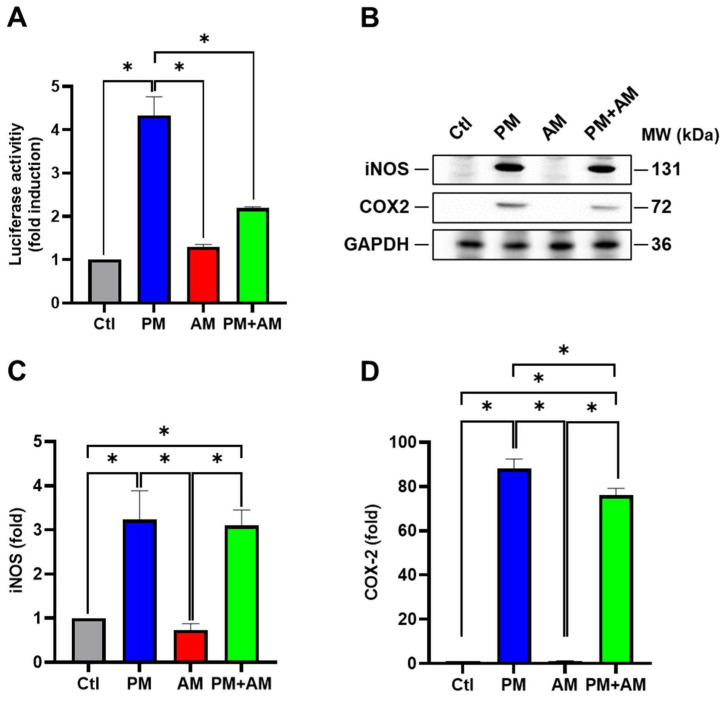
*A. mellea* mycelia mitigate NF-κB activation upon PM2.5 exposure in macrophages. (**A**) RAW264.7 cells transfected with NF-κB luciferase reporter and β-galactosidase expression vector were treated with *A. mellea* mycelial extract (200 μg/mL) and concurrently exposed to PM2.5 (30 μg/mL) for 24 h. NF-κB luciferase activity was measured and normalized to the expression level of β-galactosidase. (**B**) Cell lysates were prepared to assess the expression levels of iNOS and COX-2 using a Western blot assay. Protein expression levels of (**C**) iNOS and (**D**) COX-2 were quantified and normalized to β-actin, respectively. The data were presented as means ± standard deviations obtained from three independent experiments. Statistical significance was determined using one-way ANOVA with Tukey’s post hoc test. *, *p* < 0.05.

**Figure 3:**
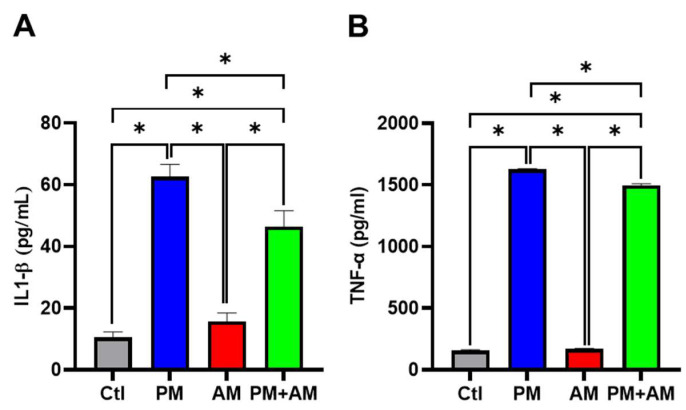
*Armillaria mellea* mycelia reduce PM2.5-induced inflammatory mediators in macrophages. RAW264.7 cells were pretreated with *A. mellea* mycelial extract (200 μg/mL) before exposure to PM2.5 (30 μg/mL) for 24 h. The secretion levels of (**A**) IL-1β and (**B**) TNF-α in the culture supernatant were quantified using an ELISA. The data were expressed as the mean ± standard deviation obtained from three independent experiments. Statistical significance was assessed using one-way ANOVA with Tukey’s post hoc test. *, *p* < 0.05.

Next, we evaluated the potential PM2.5-induced NF-κB activation by *A. mellea* mycelia using an NF-κB luciferase activity assay. Our results demonstrated that treating macrophages with *A. mellea* mycelial extract resulted in reduced NF-κB activation (Figure 2A). Since NF-κB is a crucial transcription factor responsible for initiating iNOS and COX-2 expression, we further evaluated the corresponding protein expression levels using Western blotting (Figure 2B). The quantification results revealed a significant increase in iNOS (Figure 2C) and COX-2 (Figure 2D) protein levels in cells exposed to PM2.5, which were markedly decreased following treatment with *A. mellea* mycelial extract. In addition, we observed a notable suppression of the proinflammatory cytokines, IL-1β and TNF-α, in macrophages treated with the *A. mellea* mycelial extract (Figure 3). In contrast, the phagocytic activity of the macrophages remained unaffected by treatment with *A. mellea* mycelial extract (Figure S3). These findings strongly suggest that *A. mellea* mycelia possess potent properties for suppressing the PM2.5-induced production of inflammatory mediators.

### 3.3. A. mellea Mycelia Relieve Pulmonary Inflammation in Long-Term PM2.5 Exposure in Murine Models

**Figure 4:**
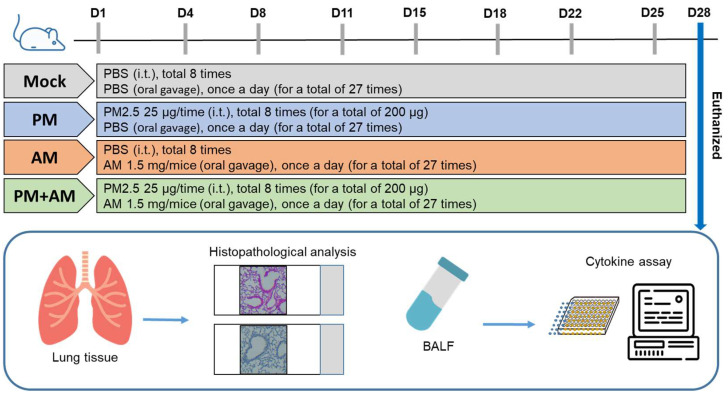
Experimental design and establishment of murine models to evaluate the biological effects of *A. mellea*. Mice received intratracheal administration of PM2.5 from day 1 to day 25, totaling 200 μg. Simultaneously, mice were treated with *A. mellea* mycelial extract (1.5 mg/mouse) once daily for a total of 27 days. Upon euthanasia of the mice on day 28, bronchoalveolar lavage fluid (BALF) was collected for cytokine analysis, and lung tissues were prepared for histopathological examination.

**Figure 5:**
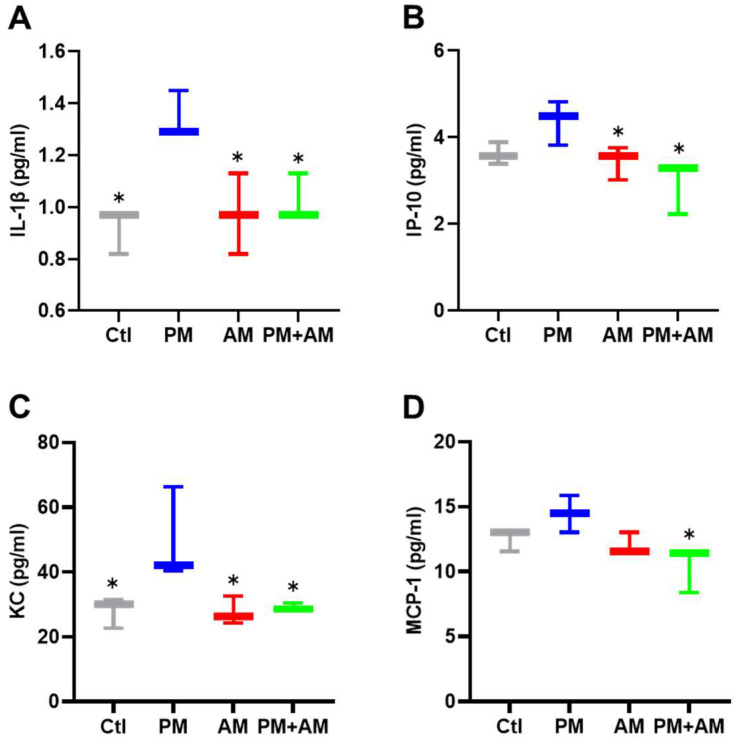
*A. mellea* mycelia alleviate PM2.5-induced inflammatory mediators in mice. Mice were administered PM2.5 and treated with *A. mellea* mycelial extract according to the experimental design in Figure 4. Following euthanasia, BALF collected from the mice was subjected to an ELISA to measure the concentrations of (**A**) IL-1β, (**B**) IP-10, (**C**) KC, and (**D**) MCP-1. The data are presented as the mean ± standard deviation obtained from each treatment group. Statistical significance was determined using the Mann–Whitney U test. *, *p* < 0.05.

**Figure 6:**
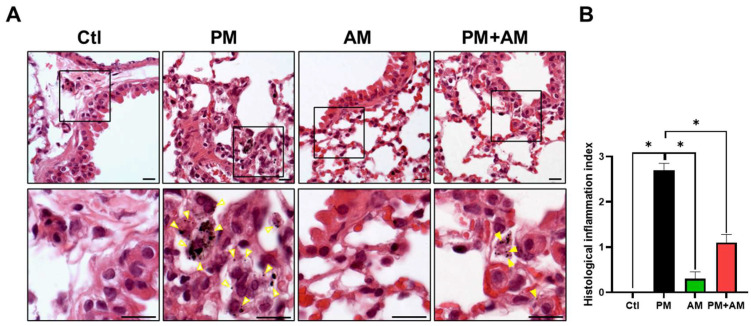
*A. mellea* mycelia attenuate PM2.5-induced lung tissue inflammation in murine models. Mice were administered PM2.5 and treated with *A. mellea* mycelial extract following the protocol outlined in Figure 4. (**A**) Upon euthanasia, lung tissue sections were subjected to H&E staining. Magnified images are displayed beneath each corresponding cropped area. The yellow arrowheads indicate macrophages with phagocytosed PM2.5. Scale bars, 10 μm. (**B**) Inflammatory cell infiltration in the lungs was assessed using a scoring system adapted from a previous study [9]: score 0, normal lung tissue; score 1, slight inflammation; score 2, moderate inflammation; score 3, severe inflammation. Statistical significance was determined using the Mann–Whitney U test. *, *p* < 0.05.

To evaluate the potential of *A. mellea* mycelial extract to ameliorate PM2.5-induced inflammation and pathogenesis in the host’s respiratory tract, we established a panel of long-term PM2.5-exposed murine models (Figure 4). We divided the mice into four groups as follows: (i) untreated mock, (ii) PM2.5-exposed, (iii) *A. mellea* mycelial extract-treated, and (iv) PM2.5-exposed + *A. mellea* mycelial extract-treated. We exposed the mice to PM2.5 for 25 d (totaling 200 μg) and simultaneously administered *A. mellea* mycelial extract (1.5 mg/mouse) once daily for 27 d. On day 28, we euthanized the mice and harvested their bronchoalveolar lavage fluid (BALF) for cytokine analysis. We registered significantly reduced IL-1β, IP-10, KC, and MCP-1 secretion levels in the *A. mellea* mycelial extract-treated mice before PM2.5 exposure compared to those exposed to PM2.5 alone (Figure 5). Subsequently, we prepared lung tissue samples for H&E staining to assess the number of infiltrated inflammatory cells. The lung tissues isolated from the untreated mock mice exhibited healthy alveoli and bronchi with minimal inflammatory cell infiltration (Figure 6). Following exposure to PM2.5, which accumulated in the cells, the bronchial wall thickened, and inflammatory cell infiltration significantly increased compared to that in the healthy mice. Conversely, when we exposed the mice to PM2.5 and concurrently treated them with *A. mellea* mycelial extract, the treatment effectively prevented the development of an inflammatory response in their lungs. Thus, our study demonstrated that *A. mellea* mycelial extract could mitigate PM2.5-induced inflammation and pathogenesis in the murine respiratory tract.

### 3.4. A. mellea Mycelia Improve PM2.5-Induced Pulmonary Inflammation

**Figure 7:**
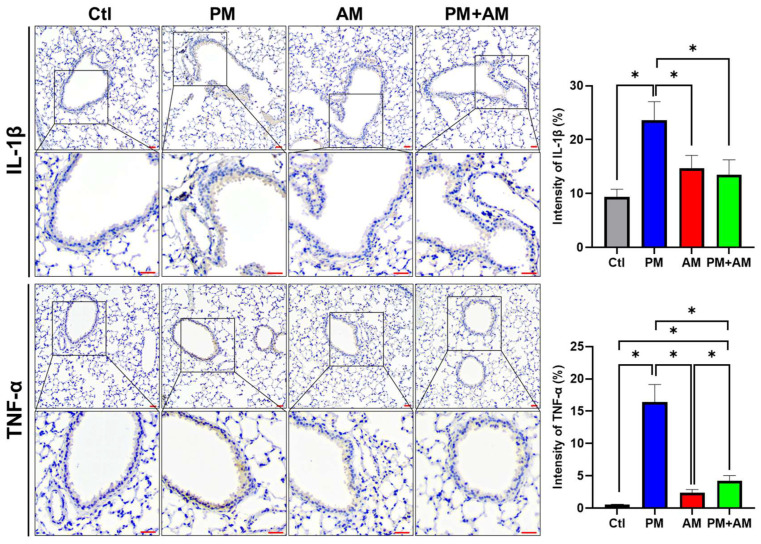
*A. mellea* mycelia decrease the expression of IL-1β and TNF-α in PM2.5-exposed lung tissues. Mice were administered PM2.5 and *A. mellea* mycelial extract following the protocol outlined in Figure 4. Murine lung tissues were prepared for immunohistochemical (IHC) staining to assess the expression levels of IL-1β and TNF-α, respectively. Magnified images are displayed beneath each corresponding cropped area. Scale bars: 200 μm. The intensity of IL-1β and TNF-α expression in gastric tissues, as detected by IHC staining, is shown in the right panels. Statistical significance was determined using the Mann–Whitney U test. *, *p* < 0.05.

**Figure 8:**
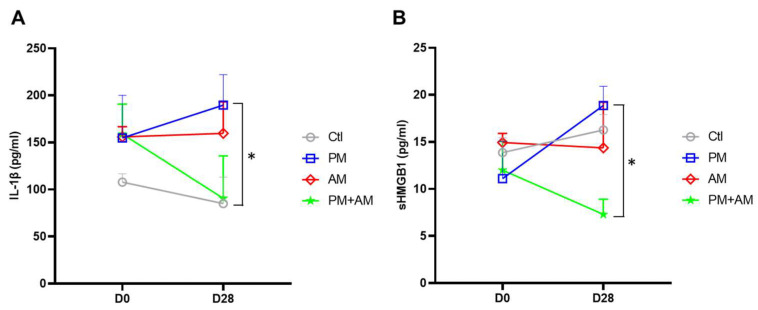
*A. mellea* mycelia mitigate PM2.5-induced inflammation in mice. Mice were administered PM2.5 and treated with *A. mellea* mycelial extract according to the protocol described in Figure 4. After euthanizing the mice, serum samples were collected on day 0 and day 28, then subjected to ELISA to measure the concentrations of (**A**) IL-1β and (**B**) sHMGB1. Statistical significance was assessed using the Mann–Whitney U test. *, *p* < 0.05.

To investigate whether *A. mellea* mycelial extract would mitigate PM2.5-induced lung inflammation, we subjected murine lung tissue samples to IHC analysis. Lung tissues isolated from the PM2.5-exposed mice exhibited a pronounced expression of proinflammatory cytokines (IL-1β and TNF-α). In the PM2.5-exposed mice treated with *A. mellea* mycelial extract, the production of these cytokines was remarkably reduced compared to that in the healthy group (Figure 7). In addition, we investigated the proinflammatory cytokine levels in the murine serum, revealing significantly increased IL-1β and sHMGB1 levels in the serum of PM2.5-exposed mice compared to their day-0 levels (Figure 8). In contrast, mice treated with *A. mellea* mycelial extract for 28 d exhibited prominently reduced IL-1β and sHMGB1 secretion levels compared to those of the PM2.5-exposed group. These results demonstrate the efficacy of *A. mellea* mycelial extract in mitigating PM2.5-induced inflammation and the related respiratory system pathogenesis, indicated by the reduced cytokine secretion and lung inflammatory status restoration in long-term PM2.5-exposed models.

## 4. Discussion

In this study, we investigated the potential of *A. mellea* mycelia to alleviate PM2.5-induced inflammation and respiratory pathogenesis. Moreover, by exploring the involved molecular mechanisms, we gained a deeper insight into how *A. mellea* mycelia mitigate PM2.5-induced respiratory inflammation and restore pulmonary function. Our results demonstrated that PM2.5 exposure impaired macrophage functions and exacerbated respiratory inflammation, which closely mirrors real-life environmental exposure scenarios. Building on this well-established platform, we employed these in vitro and in vivo models to assess the effects of *A. mellea* mycelia in alleviating PM2.5-induced macrophage inflammation. Our results emphasize the preventive potential of *A. mellea* mycelia in addressing PM2.5-related pulmonary pathogenesis.

*A. mellea*, renowned for its medicinal components such as polysaccharides and sesquiterpenes, exhibits a broad spectrum of biological activities. Polysaccharides isolated from *A. mellea* have been extensively studied and have shown notable health benefits, including immunoregulatory [28], anti-tumor [29], anti-inflammatory [30], antioxidant [31], neuroprotective [32], and lung-protective [18] properties. We used ethanol extracts of *A. mellea* mycelia in this study, assuming their richness in sesquiterpenes, as described in our recent publication [22]. Furthermore, the sesquiterpenes in *A. mellea* have been studied for their anti-tumor [33] and anti-depressant [34] effects. In conjunction with the aforementioned findings, the multifaceted biological activities of *A. mellea* mycelial sesquiterpenes support their potential as valuable therapeutic agent sources for addressing various health conditions.

*A. mellea* has been valued for its medicinal properties for centuries. In traditional medicine, such fungal extracts and preparations have been used to treat various health concerns, including respiratory ailments, gastrointestinal disorders, and neurodegenerative diseases [18,35,36]. However, *A. mellea* is a natural product with a complex chemical composition, and its efficacy may vary depending on its geographical origin, growth conditions, and extraction methods [19,20]. The standardization of *A. mellea* preparations and rigorous quality control measures are essential for ensuring the consistency and the reproducibility of therapeutic outcomes.

In this study, we established a macrophage model to investigate the effects of *A. mellea* mycelial extract on PM2.5-induced inflammation. Our findings revealed that *A. mellea* mycelial extract efficiently reduced PM2.5-triggered NO production in macrophages. We observed a concentration-dependent decrease in NO levels in *A. mellea* mycelial extract-treated macrophages, indicating the capacity of this extract to modulate the inflammatory responses initiated by PM2.5 exposure. Furthermore, *A. mellea* mycelial extract diminished NF-κB activation, leading to the downregulation of key inflammatory mediators such as iNOS and COX-2. Our findings are consistent with those of previous studies [30,35,37], demonstrating the antioxidant and anti-inflammatory properties of *A. mellea* mycelial extract, including the repression of NO production, the suppression of NF-κB signaling, and the subsequent inhibition of proinflammatory cytokine production. These results highlight the potential of *A. mellea* mycelial extract to mitigate PM2.5-induced inflammation at the molecular level.

In the murine models of long-term PM2.5 exposure, the administration of *A. mellea* mycelial extract significantly alleviated pulmonary inflammation. The reduced secretion levels of proinflammatory cytokines (IL-1β, IP-10, KC, and MCP-1) in BALF indicate the anti-inflammatory effects of *A. mellea* mycelial extract in vivo. Our histological analysis further corroborated these findings, demonstrating reduced inflammatory cell infiltration in the lungs of the *A. mellea* mycelia-treated mice compared to those in the PM2.5-exposed mice. Although the murine models of long-term PM2.5 exposure provide valuable insights into the efficacy of *A. mellea* mycelial extract in alleviating pulmonary inflammation, these models may not fully recapitulate the complexity of PM2.5 exposure-induced human respiratory diseases and the biological effects of *A. mellea*, especially in its broader roles in immune regulation, anti-inflammatory responses, and antioxidative stress. In this study, we focused on macrophages as the primary assay platform; however, we acknowledge that further research is essential to explore *A. mellea*’s interactions with other cell types and its overall health impacts. Further disease-specific clinical studies in humans should be conducted to validate our findings in a translational context.

## 5. Conclusions

In summary, our study illustrates the potent antioxidant and anti-inflammatory properties of *A. mellea* mycelia in managing PM2.5-induced inflammation and respiratory pathogenesis. In macrophage models, *A. mellea* mycelia effectively reduced NO production and suppressed NF-κB activation, thereby reducing inflammatory mediator expression. Moreover, in long-term PM2.5-exposed murine models, *A. mellea* mycelia significantly attenuated the secretion of proinflammatory cytokines and restored lung inflammatory status. The outcomes of our study hold promising potential for developing novel interventions aimed at mitigating the adverse effects of PM2.5 on respiratory health, thereby providing invaluable insights into improving human well-being.

## Data Availability

All relevant data are contained within the article. The original contributions presented in the study were included in the main article and the Supplementary Material. Further inquiries can be directed to the corresponding authors.

## Supplementary Materials

The following supporting information can be downloaded at: https://www.mdpi.com/article/10.3390/antiox13111381/s1, Figure S1. HPLC profiles of *A. mellea* mycelial extract. Figure S2. Assessment of PM2.5-induced nitric oxide production in macrophages. Figure S3. Effect of *A. mellea* mycelia on macrophage phagocytosis.

## References

[1] Feng S., Gao D., Liao F., Zhou F., Wang X. (2016). The health effects of ambient PM_2.5_ and potential mechanisms. Ecotoxicol. Environ. Saf..

[2] Riediker M., Zink D., Kreyling W., Oberdorster G., Elder A., Graham U., Lynch I., Duschl A., Ichihara G., Ichihara S. (2019). Particle toxicology and health-where are we?. Part. Fibre Toxicol..

[3] McDuffie E.E., Martin R.V., Spadaro J.V., Burnett R., Smith S.J., O’Rourke P., Hammer M.S., van Donkelaar A., Bindle L., Shah V. (2021). Source sector and fuel contributions to ambient PM_2.5_ and attributable mortality across multiple spatial scales. Nat. Commun..

[4] Guo C., Zhang Z., Lau A.K.H., Lin C.Q., Chuang Y.C., Chan J., Jiang W.K., Tam T., Yeoh E.K., Chan T.C. (2018). Effect of long-term exposure to fine particulate matter on lung function decline and risk of chronic obstructive pulmonary disease in Taiwan: A longitudinal, cohort study. Lancet Planet Health.

[5] Gehring U., Wijga A.H., Koppelman G.H., Vonk J.M., Smit H.A., Brunekreef B. (2020). Air pollution and the development of asthma from birth until young adulthood. Eur. Respir. J..

[6] Stafoggia M., Oftedal B., Chen J., Rodopoulou S., Renzi M., Atkinson R.W., Bauwelinck M., Klompmaker J.O., Mehta A., Vienneau D. (2022). Long-term exposure to low ambient air pollution concentrations and mortality among 28 million people: Results from seven large european cohorts within the ELAPSE project. Lancet Planet. Health.

[7] Xin D., Xin L. (2022). The impact of economic policy uncertainty on PM_2.5_ pollution-evidence from 25 countries. Environ. Sci. Pollut. Res. Int..

[8] Zhang J., Zeng X., Li Y., Zhao W., Chen Z., Du Q., Zhou F., Ji N., Huang M. (2019). Exposure to Ambient Particles Alters the Evolution of Macrophage Phenotype and Amplifies the Inducible Release of Eotaxin-1 in Allergen-Sensitized Mice. J. Biomed. Nanotechnol..

[9] Chen Y.W., Huang M.Z., Chen C.L., Kuo C.Y., Yang C.Y., Chiang-Ni C., Chen Y.M., Hsieh C.M., Wu H.Y., Kuo M.L. (2020). PM_2.5_ impairs macrophage functions to exacerbate pneumococcus-induced pulmonary pathogenesis. Part. Fibre Toxicol..

[10] Su R., Jin X., Zhang W., Li Z., Liu X., Ren J. (2017). Particulate matter exposure induces the autophagy of macrophages via oxidative stress-mediated PI3K/AKT/mTOR pathway. Chemosphere.

[11] Hou T., Zhu L., Wang Y., Peng L. (2024). Oxidative stress is the pivot for PM_2.5_-induced lung injury. Food Chem. Toxicol..

[12] Chen J., Braun D., Christidis T., Cork M., Rodopoulou S., Samoli E., Stafoggia M., Wolf K., Wu X., Yuchi W. (2023). Long-Term Exposure to Low-level PM_2.5_ and Mortality: Investigation of Heterogeneity by Harmonizing Analyses in Large Cohort Studies in Canada, United States, and Europe. Environ. Health Perspect..

[13] Lin C.M., Huang T.H., Chi M.C., Guo S.E., Lee C.W., Hwang S.L., Shi C.S. (2022). N-acetylcysteine alleviates fine particulate matter (PM_2.5_)-induced lung injury by attenuation of ROS-mediated recruitment of neutrophils and Ly6C(high) monocytes and lung inflammation. Ecotoxicol. Environ. Saf..

[14] Ren X., Tang Y., Sun J., Feng J., Chen L., Chen H., Zeng S., Chen C., Li X., Zhu H. (2018). Flavone protects HBE cells from DNA double-strand breaks caused by PM_2.5_. Hum. Cell.

[15] Kim J.M., Kang J.Y., Park S.K., Moon J.H., Kim M.J., Lee H.L., Jeong H.R., Kim J.C., Heo H.J. (2021). Powdered Green Tea (Matcha) Attenuates the Cognitive Dysfunction via the Regulation of Systemic Inflammation in Chronic PM_2.5_-Exposed BALB/C Mice. Antioxidants.

[16] Lee W., Ku S.K., Kim J.E., Cho S.H., Song G.Y., Bae J.S. (2019). Inhibitory Effects of Black Ginseng on Particulate Matter-Induced Pulmonary Injury. Am. J. Chin. Med..

[17] Han B.H., Jang S.H., Jang Y.J., Na S.W., Yoon J.J., Moon H.G., Kim S.Y., Seo C.S., Lee H.S., Lee Y.M. (2023). Diesel vehicles-derived PM_2.5_ induces lung and cardiovascular injury attenuates by Securiniga suffruticosa: Involvement of NF-kappaB-mediated NLRP3 inflammasome activation pathway. Biomed. Pharmacother..

[18] Chen X., Liu Y., Ren L., Dai X., Zhao J., Gao C., Zhang S., Dong J., Zhao Z., Li Y. (2024). Extraction, purification, structural characteristics and biological properties of the polysaccharides from *Armillaria mellea* (Vahl) P. Kumm.: A review. Int. J. Biol. Macromol..

[19] Kostic M., Smiljkovic M., Petrovic J., Glamoclija J., Barros L., Ferreira I., Ciric A., Sokovic M. (2017). Chemical, nutritive composition and a wide range of bioactive properties of honey mushroom *Armillaria mellea* (Vahl: Fr.) Kummer. Food Funct..

[20] Erbiai E.H., da Silva L.P., Saidi R., Lamrani Z., Esteves da Silva J.C.G., Maouni A. (2021). Chemical Composition, Bioactive Compounds, and Antioxidant Activity of Two Wild Edible Mushrooms *Armillaria mellea* and *Macrolepiota procera* from Two Countries (Morocco and Portugal). Biomolecules.

[21] Mwangi R.W., Macharia J.M., Wagara I.N., Bence R.L. (2022). The antioxidant potential of different edible and medicinal mushrooms. Biomed. Pharmacother..

[22] Li I.C., Lin T.W., Lee T.Y., Lo Y., Jiang Y.M., Kuo Y.H., Chen C.C., Chang F.C. (2021). Oral Administration of *Armillaria mellea* Mycelia Promotes Non-Rapid Eye Movement and Rapid Eye Movement Sleep in Rats. J. Fungi.

[23] Klouda G.A., Filliben J.J., Parish H.J., Chow J.C., Watson J.G., Cary R.A. (2005). Reference material 8785: Air particulate matter on filter media. Aerosol Sci Technol..

[24] Lin H.J., Jiang Z.P., Lo H.R., Feng C.L., Chen C.J., Yang C.Y., Huang M.Z., Wu H.Y., Chen Y.A., Chen Y. (2019). Coalescence of RAGE in Lipid Rafts in Response to Cytolethal Distending Toxin-Induced Inflammation. Front. Immunol..

[25] Lin C.D., Kou Y.Y., Liao C.Y., Li C.H., Huang S.P., Cheng Y.W., Liao W.C., Chen H.X., Wu P.L., Kang J.J. (2014). Zinc oxide nanoparticles impair bacterial clearance by macrophages. Nanomedicine.

[26] Chen Y.H., Tsai W.H., Wu H.Y., Chen C.Y., Yeh W.L., Chen Y.H., Hsu H.Y., Chen W.W., Chen Y.W., Chang W.W. (2019). Probiotic *Lactobacillus* spp. Act against *Helicobacter pylori*-induced Inflammation. J. Clin. Med..

[27] Lai C.H., Lin T.L., Huang M.Z., Li S.W., Wu H.Y., Chiu Y.F., Yang C.Y., Chiu C.H., Lai C.H. (2022). Gut Commensal *Parabacteroides goldsteinii* MTS01 Alters Gut Microbiota Composition and Reduces Cholesterol to Mitigate *Helicobacter pylori*-Induced Pathogenesis. Front Immunol..

[28] Sun Y., Liang H., Zhang X., Tong H., Liu J. (2009). Structural elucidation and immunological activity of a polysaccharide from the fruiting body of *Armillaria mellea*. Bioresour. Technol..

[29] Wu J., Zhou J., Lang Y., Yao L., Xu H., Shi H., Xu S. (2012). A polysaccharide from *Armillaria mellea* exhibits strong in vitro anticancer activity via apoptosis-involved mechanisms. Int. J. Biol. Macromol..

[30] Chang C.W., Lur H.S., Lu M.K., Cheng J.J. (2013). Sulfated polysaccharides of *Armillariella mellea* and their anti-inflammatory activities via NF-κB suppression. Food Res. Int..

[31] Prasad R., Varshney V.K., Harsh N.S., Kumar M. (2015). Antioxidant Capacity and Total Phenolics Content of the Fruiting Bodies and Submerged Cultured Mycelia of Sixteen Higher Basidiomycetes Mushrooms from India. Int. J. Med. Mushrooms.

[32] Rai S.N., Mishra D., Singh P., Vamanu E., Singh M.P. (2021). Therapeutic applications of mushrooms and their biomolecules along with a glimpse of in silico approach in neurodegenerative diseases. Biomed. Pharmacother..

[33] Chen C.C., Kuo Y.H., Cheng J.J., Sung P.J., Ni C.L., Chen C.C., Shen C.C. (2015). Three New Sesquiterpene Aryl Esters from the Mycelium of *Armillaria mellea*. Molecules.

[34] Zhang T., Du Y., Liu X., Sun X., Cai E., Zhu H., Zhao Y. (2021). Study on antidepressant-like effect of protoilludane sesquiterpenoid aromatic esters from *Armillaria mellea*. Nat. Prod. Res..

[35] Geng Y., Zhu S., Cheng P., Lu Z.M., Xu H.Y., Shi J.S., Xu Z.H. (2017). Bioassay-guided fractionation of ethyl acetate extract from *Armillaria mellea* attenuates inflammatory response in lipopolysaccharide (LPS) stimulated BV-2 microglia. Phytomedicine.

[36] Yao L., Lv J., Duan C., An X., Zhang C., Li D., Li C., Liu S. (2022). *Armillaria mellea* fermentation liquor ameliorates p-chlorophenylalanine-induced insomnia associated with the modulation of serotonergic system and gut microbiota in rats. J. Food Biochem..

[37] Chang C.C., Cheng J.J., Lee I.J., Lu M.K. (2018). Purification, structural elucidation, and anti-inflammatory activity of xylosyl galactofucan from *Armillaria mellea*. Int. J. Biol. Macromol..

