# Supplementary material for: Armillaria mellea Mycelia Alleviate PM2.5-Induced Pulmonary Inflammation in Murine Models
Source: Antioxidants (Basel). 2024 Nov 12;13(11):1381. doi: 10.3390/antiox13111381 (PMC11590969; doi:10.3390/antiox13111381)
Supplement: Supplementary file 1 [file antioxidants-13-01381-s001.zip › antioxidants-3170025-supplementary.pdf]

antioxidants

Supplementary Figures

Armillaria mellea Mycelia Alleviate PM2.5-Induced Pulmonary Inflammation in Murine Models

Yi-Ping Huang [1], Yu-Tsen Huang [2,3], Hui-Yu Wu [3], Li-Fang Chou [3,4], You-Shan Tsai [5], Yih-Min Jiang [5], Wan-Ping Chen [5], Ting-Wei Lin [5], Chin-Chu Chen [5,6,7,8,*] and Chih-Ho Lai [2,3,9,10,11,*]

1 Department of Physiology, School of Medicine, China Medical University, Taichung 404333, Taiwan
2 Department of Microbiology and Immunology, School of Medicine, China Medical University, Taichung 404333, Taiwan
3 Department of Microbiology and Immunology, Graduate Institute of Biomedical Sciences, Chang Gung University, Taoyuan 333323, Taiwan
4 Kidney Research Center, Chang Gung Memorial Hospital at Linkou, Taoyuan 333423, Taiwan
5 Biotech Research Institute, Grape King Bio, Taoyuan 325002, Taiwan
6 Institute of Food Science and Technology, National Taiwan University, Taipei 106216, Taiwan
7 Department of Food Sciences, Nutrition, and Nutraceutical Biotechnology, Shih Chien University, Taipei 104336, Taiwan
8 Department of Bioscience Technology, Chung Yuan Christian University, Taoyuan 320314, Taiwan
9 Department of Nursing, Asia University, Taichung 413305, Taiwan
10 Research Center for Emerging Viral Infections, Institute of Immunology and Translational Medicine, Chang Gung University, Taoyuan 333323, Taiwan
11 Molecular Infectious Disease Research Center, Chang Gung Memorial Hospital at Linkou, Taoyuan 333423, Taiwan
* Correspondence: gkbioeng@grapeking.com.tw (C.-C.C.); chlai@mail.cgu.edu.tw (C.-H.L.)

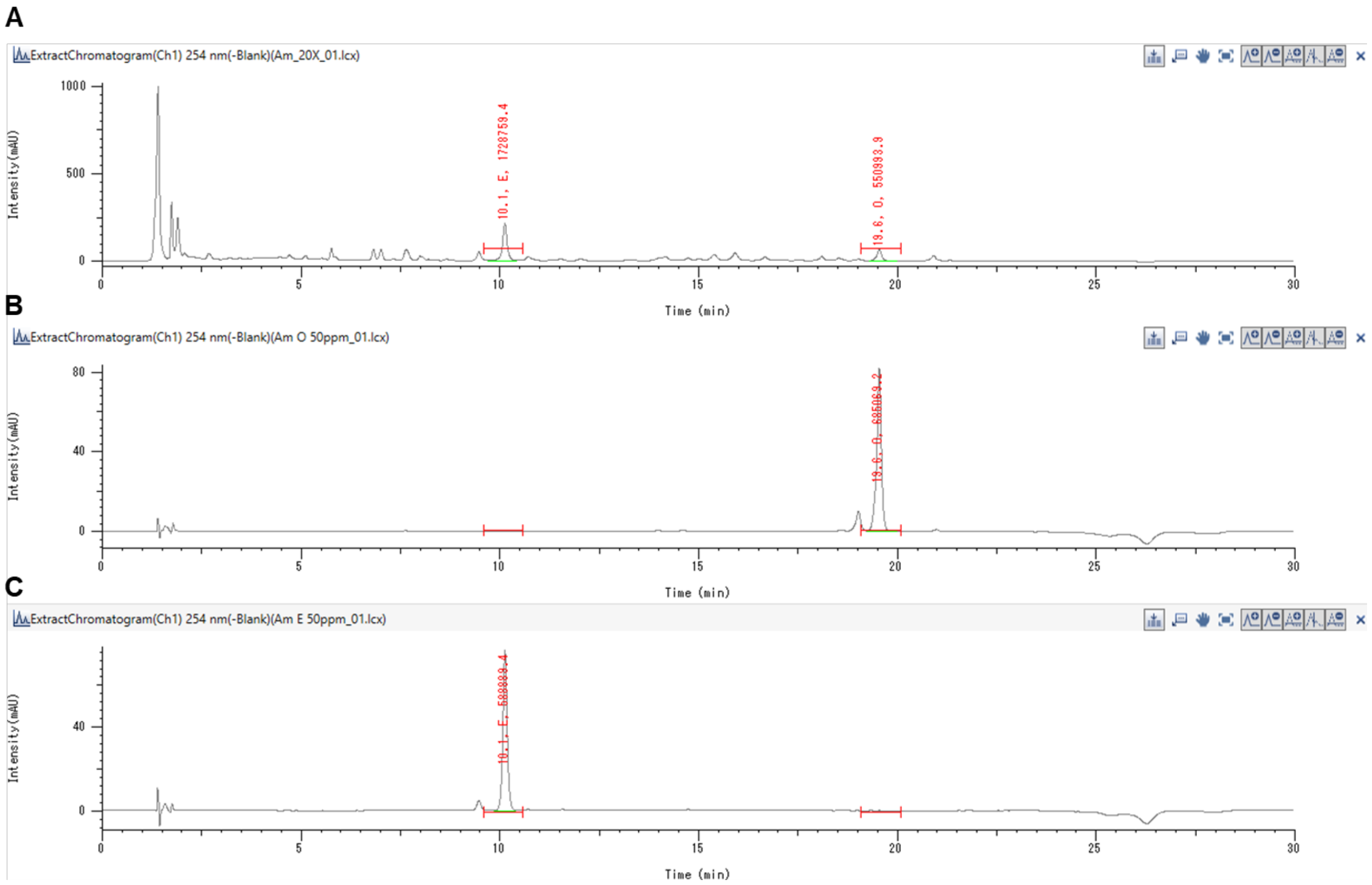

Figure S1. HPLC profiles of *A. mellea* mycelial extract. (A) HPLC analysis was performed to identify and characterize the key components of *A. mellea* mycelial extract. The standard compounds (B) Armillaridin (retention time: 19.6 min) and (C) Melledonal C (retention time: 10.1 min) were analyzed using HPLC.

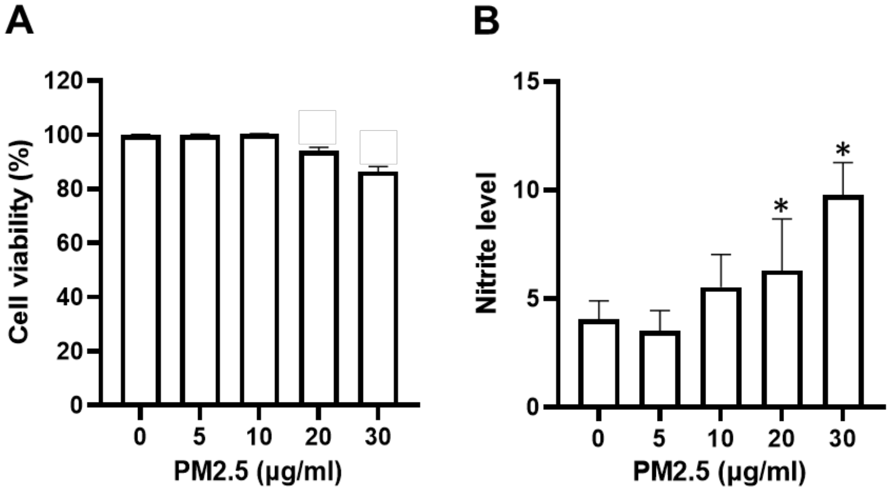

Figure S2. Assessment of PM2.5-induced nitric oxide production in macrophages. RAW264.7 cells were treated with PM2.5 at the indicated concentrations for 24 h. (A) Cell viability was assessed using the MTT assay. (B) Nitric oxide production in the culture supernatant was quantified using Griess reagent. The data are presented as the mean ± standard deviation from three independent experiments. Statistical significance was performed by one-way ANOVA followed by a post-hoc test. *, $p < 0.05$.

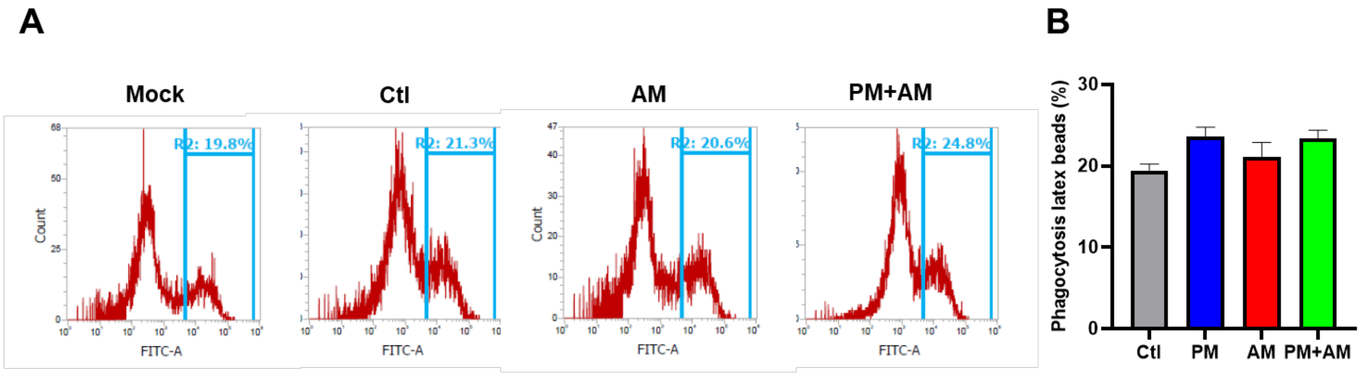

Figure S3. Effect of *A. mellea* mycelia on macrophage phagocytosis. RAW264.7 cells were pretreated with *A. mellea* mycelial extract (200 µg/mL) followed by exposure to PM2.5 (30 µg/mL) for 24 h. (A) The fluorescent intensity of latex beads coated with fluorescent-labeled rabbit IgG was analyzed using flow cytometry. (B) Quantification of the fluorescent intensity was performed. The data are expressed as means ± standard deviations obtained from three independent experiments. Statistical significance was assessed using one-way ANOVA followed by a post-hoc test. *, $p < 0.05$.
